# Neurological examination of healthy term infants at ages 6 and 10 weeks in Tshwane District

**DOI:** 10.4102/sajp.v80i1.2072

**Published:** 2024-08-30

**Authors:** Marna Nel, Ute Feucht, Helen Mulol, Carina A. Eksteen

**Affiliations:** 1 Research Centre for Maternal, Fetal, Newborn and Child Health Care Strategies, Faculty of Health Science, University of Pretoria, Pretoria, South Africa; 2 Maternal and Infant Health Care Strategies Research Unit, South African Medical Research Council, Pretoria, South Africa; 3 Department of Paediatrics, Faculty of Health Sciences, University of Pretoria, Pretoria, South Africa; 4 Department of Physiotherapy, School of Health Care Sciences, Sefako Makgatho Health Science University, Pretoria, South Africa

**Keywords:** infants, Hammersmith Neonatal Neurological Examination, neurodevelopment, optimality scores, milestone characteristics, Sustainable Developmental Goals

## Abstract

**Background:**

Globally, there is a significant gap in detailed neurodevelopmental data for infants under 3 months, despite 6 weeks being identified a critical milestone for neuro-behavioural development. Normative values and optimal scores for healthy infants at 6 and 10 weeks postnatally are lacking in many settings. In South Africa, the statutory neurodevelopmental assessments at these ages exclude notable characteristics of central nervous system maturation and limit opportunities to collect data of early developmental progress.

**Objectives:**

Our study aimed to assess developmental characteristics of healthy term infants aged 6 and 10 weeks using the Hammersmith Neonatal Neurological Examination (HNNE).

**Method:**

A prospective longitudinal study was performed on 35 healthy term-born infants from low-risk pregnancies at 6 and 10 weeks’ postnatal age in the Tshwane district. The statuses of infants’ neurodevelopment in six domains were recorded using the HNNE. Optimality scores were derived from the raw scores of 34 items, using the 10th and 5th percentiles as cut-off points.

**Results:**

Evidences of neurodevelopmental advancements, particularly in posture, muscle tone and visual behaviour between 6 and 10 weeks were illustrated, and total examination optimality scores of 29.5 in 91% and 31.5 in 94% of infants were recorded at 6 and 10 weeks, respectively.

**Conclusion:**

This article provides data on the neurodevelopment characteristics of infants at and between 6- and 10-weeks post term ages.

**Clinical Implications:**

The findings support the viewpoint to identify important milestone characteristics during early screening.

## Introduction

Neurodevelopmental progress in early infancy, especially under the age of 3 months, remains globally poorly documented. Developing countries face significant, yet amendable maternal and foetal risk factors such as maternal infections, malaria, lifestyle and nutritional deficiencies (Gardosi et al. 2013; Lawn et al. 2016; Nkosi et al. 2019; Ravula et al. 2022). Adverse factors frequently co-exist and can result in conditions like placental insufficiency, which is recognised as a significant underlying cause of stillbirth and co-morbidities such as foetal growth restriction, preterm labour and neonatal encephalopathy (Bukowski et al. 2003; Hertting et al. 2023; Lawn et al. 2016; Ravula et al. 2022; Tann et al. 2018a; Tunc et al. 2022; Vik et al. 2018). These conditions can impair infants’ brain development and potentially lead to neurodevelopmental delay in surviving infants (Gardella et al. 2021; Lawn et al. 2016; Mwaniki et al. 2012; Romeo et al. 2020; Tann et al. 2018a; Tuiskula et al. 2022).

Furthermore, in developing countries, a significant number of children fail to achieve their developmental potential, necessitating early identification and intervention for at-risk infants to mitigate adverse developmental outcomes (Black et al. 2017; Colella et al. 2018; Grantham-McGregor et al. 2007; Novak et al. 2017; Tann et al. 2018b). The importance of monitoring neurological maturation during the early window periods of infants’ brain development is widely encouraged (Black 2017; Blakstad et al. 2019; Hartkopf et al. 2018; Novak 2017; Richter et al. 2017; Tann 2018b; Tomlinson et al. 2019). Guzzetta et al. (2005) noted significant changes in the developmental trajectory of muscle tone, posture and visual behaviour in healthy term infants between birth and 10 weeks, identifying the 6-week age as an important milestone for developmental screening; this study, however, was descriptive in nature and the data was not quantified. Despite the importance of early identification, there is a lack of publications providing standardised normative values and detailed milestone descriptions specifically for infants at 6- and 10-week post-term ages, thus underscoring the urgency to revise existing assessment tools to better accommodate the diverse settings found in developing countries (Faruk et al. 2020).

In South Africa, healthcare professionals use the ‘Road-to-Health Booklet’ (RtHB) to document and monitor child growth, immunisation, and developmental progress during routine postnatal follow-up visits at primary healthcare facilities (NDoH 2020). This document starts detailing developmental characteristics in a checklist manner from 14 weeks post-birth. However, the RtHB omits early neurodevelopmental milestones for infants aged 6 and 10 weeks in key areas like hearing and/or communication, vision and/or visual adaptation, cognition and/or behaviour, and motor skills. It also lacks a provision for measuring and documenting head circumference, an essential aspect of any neurological and neurodevelopmental assessment (Harris 2015; NDoH 2020; Tal et al. 2012). This oversight renders the RtHB insufficient for early identification of infants at developmental risk within the South African context (Van der Linde et al. 2015).

The Hammersmith Neonatal Neurological Examination (HNNE) offers a detailed structure for evaluating infant neurodevelopment across various domains that include *posture and tone, tone patterns, reflexes, movements, abnormal signs, orientation and behaviour* during the initial 3 months after birth (Dubowitz, Dubowitz & Mercuri 1999; Dubowitz, Ricci & Mercuri 2005). These developmental domains align with those documented in the RtHB, providing a nuanced description of milestone characteristics in its proforma evaluation form. However, the HNNE optimality scores derived from healthy term infants in a higher-income setting may not directly apply to infants in developing countries because of demonstrated differences in the newborn period (Dubowitz, Mercuri & Dubowitz 1998; Hagmann et al. 2015; Lawford et al. 2020, 2021a; McGready et al. 2000). Differences in raw score distribution, median scores, and sub-domain scores were observed even among developing countries because of various factors specific and unique to each country and the exact timeframe of the research. Some contributing factors include potential inaccuracies in calculating the mean gestational age at birth, variations in the postnatal window period from birth to 48 h, inconsistent test environments, mode of delivery, use of anaesthetics and the proficiency and inter-rater reliability of scorers. (Lawford et al. 2020). Differences in item scores ranged across all six domains such as tone, reflexes, quality of movement, visual and auditory behaviour, and abnormal signs have been reported (Dubowitz et al. 1998; Hagmann et al. 2015; Lawford et al. 2020, 2021a; McGready et al. 2000).

Despite these challenges, the HNNE remains a valuable tool for recording ranges of developmental characteristics in low-risk infants in various developing countries such as Ghana, Uganda and Vietnam, providing these countries with benchmark data and structured guidance to monitor neurodevelopment (Hagmann et al. 2015; Hieu et al. 2006; Lawford et al. 2021a). The HNNE has observed developmental variations in at-risk neonatal groups from both developing and high-income nations, and its application in several developing countries has spurred additional research aimed at establishing consistent normative and longitudinal data across diverse populations (Chin et al. 2019; Guzzetta et al. 2005; Lawford et al. 2021b; Ong et al. 2023; Spittle et al. 2016; Tuhkanen et al. 2019; Tuiskula et al. 2022; Venkata et al. 2020).

Normative HNNE optimality scores exist for the neonatal period (first 4 weeks) in countries such as the United Kingdom (UK), the Netherlands, Italy, Singapore and Australia, highlighting the need for local and global data on milestone characteristics at 6- and 10-weeks to aid early neurodevelopmental assessment and intervention (Abdoola et al. 2023; Dubowitz et al. 1998; Guzzetta et al. 2005; Mercuri et al. 2003; Novak et al. 2017; Ong et al. 2023; Ricci et al. 2008; Romeo et al. 2013, 2020; Spittle et al. 2016).

The primary aim of our study was to assess and allocate raw scores (RSs) to the developmental characteristics of healthy 6- and 10-week term infants in Tshwane District, South Africa, using the HNNE tool (Dubowitz et al. 1999). Our secondary aim was to apply the HNNE optimality scoring system to the RSs using 10th and 5th percentiles as cut-off points. This data will contribute towards documenting and understanding early infant neurodevelopment in a developing country (Dubowitz et al. 1998). To our knowledge, this is the first study on infants in this age group using the HNNE in South Africa.

## Research methods and design

### Study design

A prospective, longitudinal descriptive study was undertaken to assess and allocate RSs to the neuro-behavioural characteristics of 6- and 10-week-old infants in Tshwane district, South Africa using a standardised neurological examination method requiring direct interaction with the infant. An optimality scoring system was applied to convert the frequency distribution of the RSs to optimality scores for all the items in the proforma evaluation form and to calculate the total examination optimality scores for both age groups in this cohort.

### Setting

Our study was part of and conducted parallel to the larger UmbiBaby study investigating early childhood growth and developmental outcomes. The mother-infant dyads in this sub-study who formed part of an ongoing ante-, peri-, and postnatal investigation were followed up at their 6- and 10-week routine postnatal clinic visits at the Research Centre for Maternal, Fetal, Newborn and Child Health Care Strategies at Kalafong Hospital, Tshwane District, South Africa.

### Study population and sampling strategy

For the infant follow-up study, mothers in Tshwane district who were part of an antenatal study were approached around the time of birth at Pretoria West Hospital, Laudium Community Health Centre, and Kalafong Provincial Tertiary Hospital.

A total of 81 term-born infants were enrolled for the prospective study following growth and development of infants from 6 weeks until 2 years (the UmbiBaby Study). Written informed consent was given by the mother on behalf of the mother-infant dyad. The inclusion criteria were infants: (1) from mothers with singleton pregnancies; (2) born to mothers with low-risk pregnancies according to local antenatal care guidelines (National Department of Health 2017), (3) with known gestational ages, calculated during the antenatal screening; (4) who attended both the 6- and 10-week clinic visits. The exclusion criteria were infants: (1) born to mothers younger than 18 years of age; (2) with chromosomal and/or congenital abnormalities or malformation, and (3) severe medical conditions. A cohort of 35 infants (19 males and 16 females) who were part of the postnatal study were included in this investigation since they attended both the 6- and 10-week visits. All 35 mother-infant dyads met the inclusion criteria.

### Data collection

Our study utilised the HNNE to collect data, scoring the performance of 34 neurological items across 35 healthy term-born infants (Dubowitz et al. 1999). Each evaluation, aligned with the standard HNNE proforma, was documented on a new form for each visit; results were organised by age group. Data collection occurred during the routine 6- and 10-week postnatal clinic visits between October 2019 and June 2020, incorporating demographic and nutritional details of the mother-infant dyads as part of the broader parent postnatal study.

**FIGURE 1:**
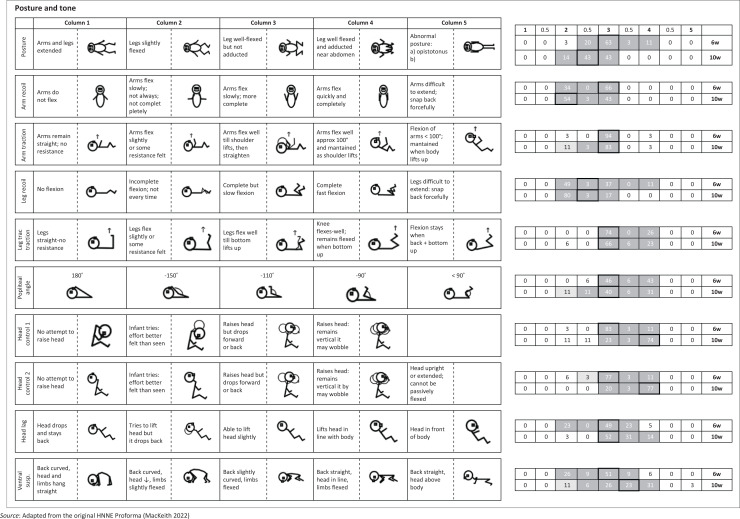
Distribution of ***posture and tone*** raw scores in the Hammersmith Neonatal Neurological Examination. Optimal scores (≥ 10th percentile) are shown in dark grey on small right-side tables, scoring 1. Borderline scores (≥ 5th – < 10th percentile) in light grey, score 0.5. Sub-optimal scores (< 5th percentile) outside grey areas score 0. Cells with highlighted borders indicate median scores.

**FIGURE 2:**
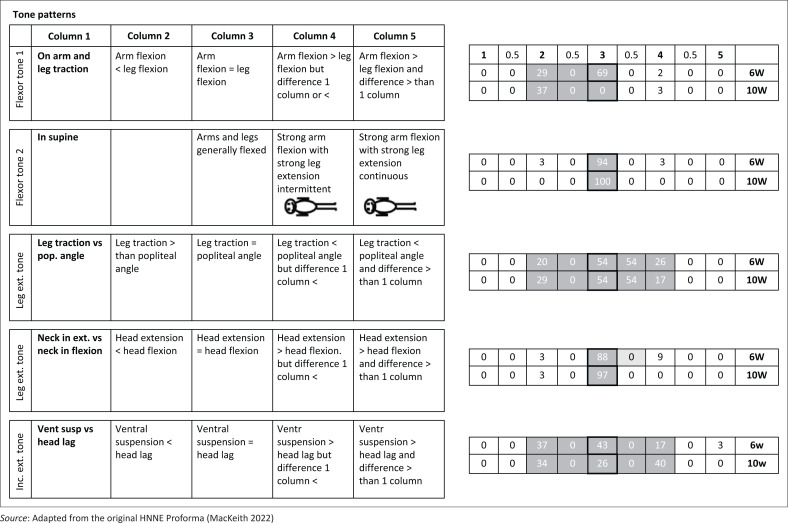
Raw score distribution of ***tone pattern*** items in the Hammersmith Neonatal Neurological Examination. Optimal scores (≥ 10th percentile) are shown in dark grey on small right-side tables, scoring 1. Borderline scores (≥ 5th – < 10th percentile) in light grey, score 0.5. Sub-optimal scores (< 5th percentile) outside grey areas score 0. Cells with highlighted borders indicate median scores.

**FIGURE 3:**
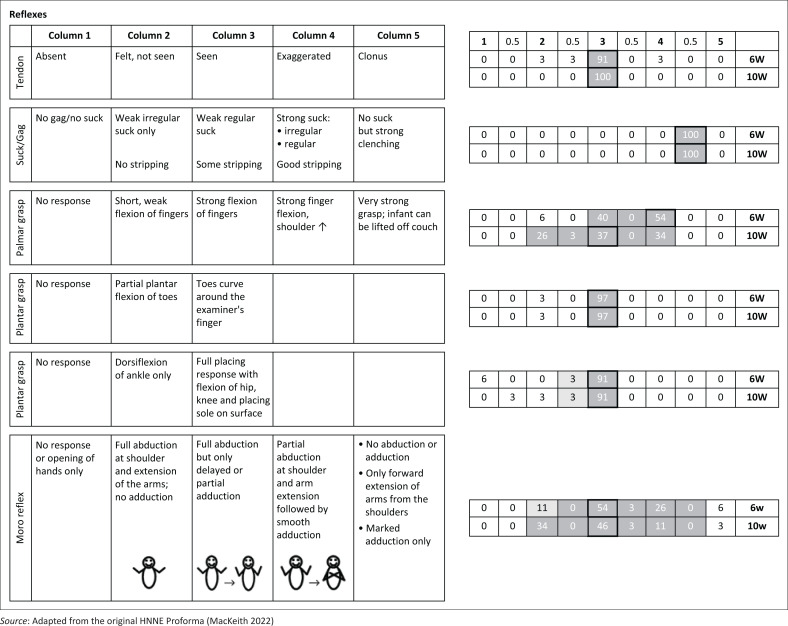
Raw score distribution of ***reflex*** items in the Hammersmith Neonatal Neurological Examination. Optimal scores (≥ 10th percentile) are shown in dark grey on small right-side tables, scoring 1. Borderline scores (≥ 5th – < 10th percentile) in light grey, score 0.5. Sub-optimal scores (< 5th percentile) outside grey areas score 0. Cells with highlighted borders indicate median scores.

**FIGURE 4:**
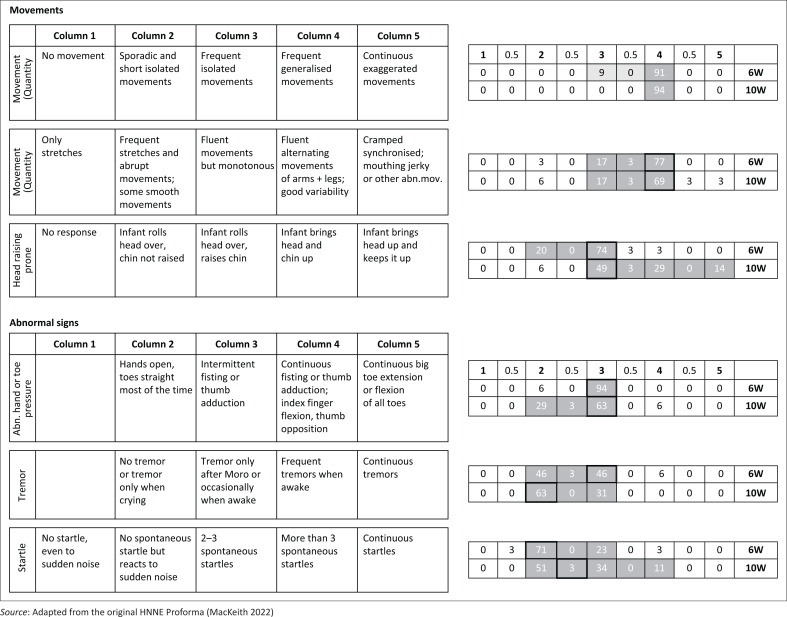
Raw score distribution of ***movement*** and ***abnormal signs*** items in the Hammersmith Neonatal Neurological Examination. Optimal scores (≥ 10th percentile) are shown in dark grey on small right-side tables, scoring 1. Borderline scores (≥ 5th – < 10th percentile) in light grey, score 0.5. Sub-optimal scores (< 5th percentile) outside grey areas score 0. Cells with highlighted borders indicate median scores.

**FIGURE 5:**
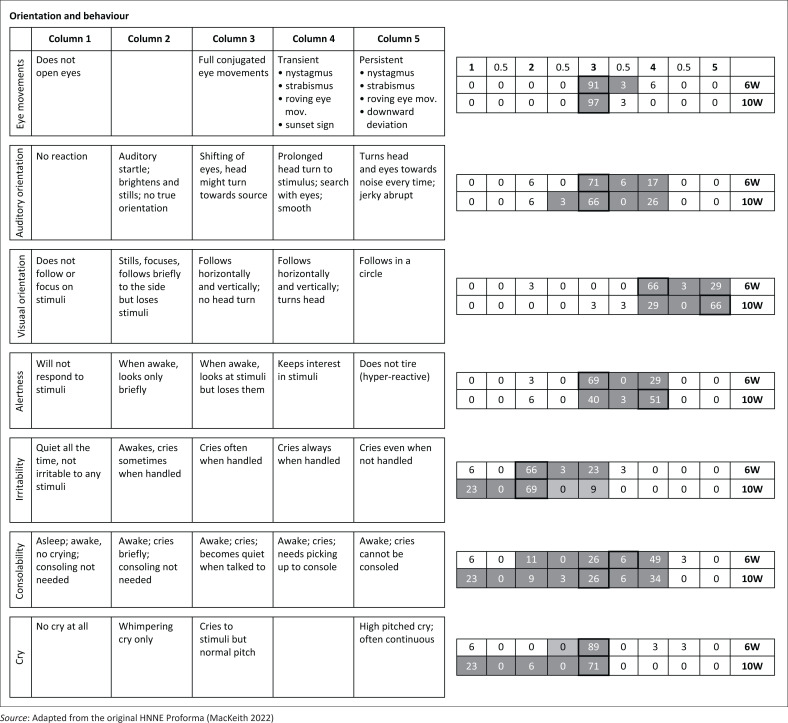
Raw score distribution of ***orientation and behaviour*** items in the Hammersmith Neonatal Neurological Examination. Optimal scores (≥ 10th percentile) are shown in dark grey on small right-side tables, scoring 1. Borderline scores (≥ 5th – < 10th percentile) in light grey, score 0.5. Sub-optimal scores (< 5th percentile) outside grey areas score 0. Cells with highlighted borders indicate median scores.

The HNNE assessments were conducted, scored and analysed by the first author, a certified physiotherapist with expertise in using and interpreting this diagnostic tool. Data were double-entered and managed on the study’s REDCap (Research Electronic Data Capture) electronic database hosted at the South African Medical Research Council. For clarity, the distribution of RSs and corresponding percentages of infants with similar scores were depicted in modified illustrations of the HNNE proforma (Figures 1–5). Optimality scores, calculated from raw data in Microsoft Excel, were presented in table format, with the 10th and 5th percentiles serving as cutoff points.

### Assessment instrument

The HNNE is a standardised assessment instrument developed to examine the neurological state of newborn term infants in the first 48 h after birth. The assessment was developed in the UK and has subsequently been used in clinical and research settings for nearly 40 years (Dubowitz et al. 1980, 1998). The assessment proforma was modified to its current form with the addition of an ‘optimality scoring system’ that was validated on 224 low-risk term infants in the UK within 48 h after birth (Dubowitz et al. 1998, 1999). To date, optimality scores have been determined and published for preterm, late preterm infant populations at term-equivalent age, and healthy term infants at 4-weeks post term age (Chin et al. 2019; Mercuri et al. 2003; Ricci et al. 2008; Romeo et al. 2013; Spittle 2016). Normative data and subsequently optimality scores have not yet been published for term infants beyond the 4-weeks post term age.

In conjunction with neuroimaging and neurophysiological techniques, the HNNE effectively identified clinical patterns associated with neurological conditions such as hypoxic ischaemic encephalopathy, periventricular leukomalacia, and intra-ventricular haemorrhage (Dubowitz et al. 1999). The examination demonstrated predictive validity in extremely preterm infants at 4 and 12 months corrected ages in correlation with outcome assessments such as Magnetic Resonance Imaging (MRI) and the Infant Neurological International Battery (INFANIB), and in low-risk preterm infants at 12 months corrected age with the Bayley Scales of Infant and Toddler Development II (Bozynski et al. 1993; Noble & Boyd 2011). Additionally, optimal neonatal HNNE scores in motor and reflex items were also predicted by foetal motor behaviour in low-risk pregnancies (DiPietro et al. 2010). The HNNE as an examination tool is suitable for clinical application with an administration time of 10 min – 15 min and interrater reliability of 96% (Dubowitz et al. 1999; Eeles et al. 2017; McGready et al. 2000). The instrument does not require formal training or certification, and can be self-taught through the Hammersmith Neurological Examination website (MacKeith 2022).

### Scoring and data analysis

The HNNE consists of 34 items that are characteristic of the functional state of the newborn infant’s central nervous system. These characteristics are categorised in six domains referred to in the HNNE proforma as *compounds*: (1) *posture and tone*, (2) *tone patterns*, (3) *reflexes*, (4) *movements*, (5) *abnormal neurological signs*, and (6) *orientation and behaviour*.

#### Raw scores

The items are scored in a standard proforma according to a five-point scale. The description of different responses and characteristics for each item are distributed horizontally over five columns. The column that represents the predominant behaviour of the infant for each item is circled and scored according to the relevant column. Half-scores are appropriate if the infant’s performance falls between the description of two columns (e.g. a score of 2.5 if the infant displays characteristics between that fall between descriptions in columns 2 and 3). These scores are defined as RSs.

The RSs are not linear and the ‘optimal score’ for each of the 34 items depends on the distribution frequency of RSs per item for the study population. For example, for the posture item, a score of 1 to 2 would indicate decreased muscle tone, a score of 3 and 4 would be normal tone and a score of 5 would indicate increased muscle tone.

The RS distribution for each item for the study population was converted to an ‘optimality score’, using the 10th and 5th percentiles as cut-off points. Items ≥ 10th percentile were given a score of 1; items ≥ 5th and < 10th percentile given a score of 0.5; and < 5th percentile were given a score of 0 (Dubowitz et al. 1999). For the infants at 6- and 10-weeks postnatal age in our study population, the same optimality scoring system was applied to their individual and collective RSs.

#### Compound scores

*Compound* scores were calculated for each of the 6 domains in the proforma: *posture and tone, tone patterns, reflexes,* spontaneous *movements, abnormal signs,* and *orientation and behaviour*. The *compound* optimality score is the sum of the optimality scores for the individual items in the domain. This score may range from 0 (if all the items in the compound are *sub-optimal*) to a maximum score equal to the number of items in the *compound* if every item is *optimal* with a score of 1 (Dubowitz et al. 1999).

#### Total examination score

The *total examination* score is the sum of the optimality scores of the 34 individual items. This score may range from 0 (if all the items were *sub-optimal*) to a maximum of 34 (if all the items were *optimal*). The range of the *compound* and *total examination* optimality scores were described as *optimal* ≥ 10th percentile, *borderline* ≥ 5th and < 10th percentile and *sub-optimal* < 5th percentile (Dubowitz et al. 1999 and see Supplementary Tables S1–S6).

### Clinical examination

The environmental conditions in which the assessment was performed were controlled for noise, bright light and temperature. Since the assessment elicits variable behavioural responses in the infants, it was performed with all infants in a baseline behavioural state 4 (quiet alert state; Brazelton & Nugent 1995). The assessment was done halfway between feeds to ensure comparable behaviour among infants. The infants were examined on a flat wooden table with a thin mat for comfort but assuring a firm and comfortable surface for bony areas, for example, the occiput when rolling the head from side to side. After uncovering the infant, only the diaper was left on; the evaluation started with a short period (2 min) of observing posture and spontaneous movement in the supine position.

The HNNE physically challenges infants and therefore the characteristics of the items such as *posture and tone* of the head, trunk and limbs in supine, and the *quality* and *quantity* of spontaneous *movement* were assessed before physical and tactile input by the examiner.

To ensure optimal performance of the infant in a quiet state, the items for *eye appearance, auditory, visual orientation* and *alertness* in the *orientation and behaviour* compound were recorded following the assessment of *posture* and spontaneous *movement*. This sequence in performing the assessment enabled the infant to complete the assessment, noting the triggers in the infant’s *irritability* and *crying* behaviour. The *Moro reflex* was elicited at the end of the assessment as infants are often startled by their own response and may then be difficult to console. The assessment ended with the measurement of the infant’s *head circumference*.

The findings for each item in all six *compounds* were recorded during the examination. The duration of the assessment was between 10 min and 15 min, but depended on the infants’ behavioural state and responses. The infants’ *consolability* (strategies required to be consoled), the intensity of the *cry* and general *irritability* (response to the handling and physical challenges of the examination) were assessed throughout the examination and were recorded last.

### Ethical considerations

Permission to conduct the study was obtained through the University of Pretoria’s Research Ethics Committee (reference number: 283/2019) as well as institutional permission from the relevant health services in the Tshwane district. The mothers of the infants in our study gave written informed consent to participate in the study as a mother-infant dyad at the first postnatal clinic visit at 6 weeks. Mothers were assigned a study number, which was used throughout the study for the purposes of anonymity.

## Results

### Bio-demographic data of the mother-infant dyads in our study population

Background, pregnancy, infant feeding and anthropometric data of the participating mother-infant dyads in our study are shown in Table 1. The mothers’ pregnancies in this cohort were considered low risk according to local antenatal care guidelines (NDoH 2017). The infants resided in formal (60%) and informal settlements (40%) in the Tshwane district. The majority (60%) were delivered by Caesarean section and 40% were delivered through normal vaginal delivery. The gestational age- and sex-normalised z-scores for weight, length and head circumference were assessed at birth using the INTERGROWTH – 21st Newborn Size at Birth standard (Villar et al. 2019). Birth measurements were done as part of the routine assessment at birth by staff members attending to the mother and infant at birth.

**TABLE 1:** Bio-demographic data of the 35 mother-infant dyads in our study population.

Variables	Birth (*N* = 35)					6 weeks (*N* = 35)			10 weeks (*N* = 35)		
	Mean ± s.d.	*n*	%	Median	Range	Mean ± s.d.	*n*	%	Mean ± s.d.	*n*	%
**Birth**											
**Infant sex, M/F§**	-	19/16	-	-	-	-	-	-	-	-	-
**Gestational age, weeks**	39.2 ± 1.4	-	-	-	-	-	-	-	-	-	-
**Mode of delivery**											
Normal vaginal delivery	-	14	40.0	-	-	-	-	-	-	-	-
Caesarean section before labour	-	6	17.1	-	-	-	-	-	-	-	-
Caesarean section during labour	-	15	42.9	-	-	-	-	-	-	-	-
**Apgar score at 5 minutes**											
0–6	-	1	2.9	-	-	-	-	-	-	-	-
7–10	-	34	97.1	-	-	-	-	-	-	-	-
**Foetal distress**	-	5	14.3	-	-	-	-	-	-	-	-
**Maternal age, years**	29.4 ± 5.7	-	-	-	-	-	-	-	-	-	-
**Gravidity**	-	-	-	2	1–5	-	-	-	-	-	-
**Maternal HIV§ infection**	-	9	25.7	-	-	-	-	-	-	-	-
**Description of neighbourhood**											
Formal settlement	-	21	60.0	-	-	-	-	-	-	-	-
Informal settlement	-	14	40.0	-	-	-	-	-	-	-	-
**Postnatal**											
Chronological age at assessment	-	-	-	-	-	6.4 ± 0.6	-	-	10.4 ± 0.6	-	-
**Measurements†**											
Weight, kg	3.11 ± 0.6	-	-	-	-	4.9 ± 0.7	-	-	5.85 ± 0.8	-	-
Head circumference, cm	34.7 ± 1.6	-	-	-	-	38.9 ± 1.4	-	-	40.5 ± 1.4	-	-
Length, cm	50.0 ± 3.0	-	-	-	-	54.8 ± 2.6	-	-	58.6 ± 2.8	-	-
**Z-scores** ‡											
Weight for age	0.24 ± 0.26	-	-	-	-	0.09 ± 1.17	-	-	0.12 ± 1.16	-	-
Head circumference for age	0.85 ± 1.21	-	-	-	-	0.73 ± 1.24	-	-	0.98 ± 1.14	-	-
Length for age	0.58 ± 1.60	-	-	-	-	0.74 ± 1.31	-	-	−0.27 ± 1.36	-	-
Weight for length	-	-	-	-	-	0.86 ± 1.33	-	-	0.61 ± 1.09	-	-
**Mode of feeding**											
Breast	-	27	77.1	-	-	-	26	74.3	-	24	68.6
Mixed	-	6	17.1	-	-	-	6	17.1	-	7	20.0
Formula	-	2	5.7	-	-	-	3	8.6	-	4	11.4

† , INTERGROWTH – 21st Newborn Size at Birth standard (Villar et al. 2019).

‡ , World Health Organization (WHO) child growth standards (De Onis et al. 2006).

§ , M/F = male/female; HIV = human immunodeficiency virus.

Weight, length and head circumference were measured by research staff, and the World Health Organization (WHO) child growth standards (De Onis et al. 2006) were used to determine the infant’s anthropometrical age- and sex-normalised Z-scores at the 6- and 10-week visits (Table 1). The mean z-scores for infant anthropometry at birth, 6 and 10 weeks all lay between −1 and +1, indicating that most of the infants fell within the range for optimal growth (i.e., between the z-scores of −2 and +2). The average chronological ages at the follow-up assessments (i.e. mean time since birth) were 6.4 and 10.4 weeks, respectively. One-four (25%) of the mothers were human immunodeficiency virus (HIV)-infected; however, none of the infants in the study tested HIV-positive.

### Clinical examination results

The 34 items of the HNNE proforma are illustrated in Figures 1–5, reflecting the six *compounds* of the examination (Dubowitz et al. 1999). The distribution of RSs for all items at 6 and 10 weeks, respectively and the percentage of infants scored in each column are illustrated by a small table on the right side of every item (Figures 1–5). The distribution of RSs was determined by the frequency in which the characteristics were displayed in each of the 34 items. The frequency of the scores that were obtained by infants in both age groups and that were considered *optimal* (≥ 10th percentile) by a score of 1, are indicated in the dark-grey areas. The light-grey columns indicate those RSs that were considered *borderline* (≥ 5th; < 10th percentile), by a score of 0.5. The RSs that fell outside the grey areas were considered *sub-optimal* (< 5th percentile) and therefore scored 0 (Figures 1–5). *Compound* scores (summation of individual item optimality scores) and the *total examination optimality* score (summation of the 34 individual item optimality scores) ranges, mean values, standard deviations, and percentages for the whole population at 6- and 10-weeks postnatal age are shown in Table 2.

**TABLE 2:** *Compound* and *total examination optimalit y* scores for 35 healthy term infants at 6- and 10-weeks post-term age included in this study.

*Compounds*	Age (weeks)	Range	Mean	s.d.	5th%	10th%	90th%	Infants with optimal scores (%)
***1. Posture and tone***	6	8–10	9.67	0.58	8.7	9.0	10.0	97
	10	8–10	9.71	0.52	8.7	9.2	10.0	94
***2. Tone patterns***	6	4–5	4.87	0.31	4.0	4.5	5.0	97
	10	4–5	4.97	0.17	5.0	5.0	5.0	97
***3. Reflexes***	6	3–6	5.64	0.72	4.0	4.7	6.0	86
	10	3–6	5.80	0.62	4.7	5.5	6.0	94
***4. Spontaneous movements***	6	1.5–3	2.87	0.35	2.0	2.5	3.0	94
	10	1–3	2.83	0.51	1.7	2.4	3.0	86
***5. Abnormal signs***	6	1–3	2.86	0.43	2.0	2.4	3.0	86
	10	2–3	2.89	0.32	2.0	2.4	3.0	86
***6. Orientation and behaviour***	6	3–7	6.54	1.09	3.7	5.4	7.0	86
	10	5–7	6.74	0.51	5.9	6.0	7.0	100
***Total examination***	6	25.5–34	32.46	2.02	28.9	29.5	34.0	91
***optimality scores***	10	29.0–34.0	32.94	1.20	30.9	31.5	34.0	94

* Suboptimal score < 5th%; Borderline score ≥ 5th and < 10th%; Optimal score ≥ 10% (Figures 1–5).

### Posture and tone

The frequency distribution of RSs in this *compound* was different for each of the 10 items at both 6 and 10 weeks (Figure 1). Median score shifts of one and two columns to the left from 6 to 10 weeks indicated characteristics of diminished flexion posture and lesser resistance of upper limbs’ response to traction and passive extension respectively (arm/leg recoil; arm/leg traction; popliteal angle). Median score shifts of one to two columns involving flexor and extensor head control in sitting and extensor control in the horizontal position occurred to the right from 6 to 10 weeks. The *compound* scores for *posture and tone* ranged from 8 to 10 out of 10 at both 6 and 10 weeks. These scores were achieved by 97% of the total population of infants at 6-weeks and 94% at 10-weeks postnatal age (Table 2).

### Tone patterns

Scores in this *compound* were obtained by comparing the RSs of selected individual items of limb tone and head control patterns from the *posture and tone compound* (Figure 2). At 6 weeks, 97% of infants scored ≥ 4.5 out of 5 and at 10 weeks 97% scored 5 out of 5 (Table 2). The distribution and median scores at 6 and 10 weeks were consistent in all five items.

### Reflexes

Palmar grasp RSs at 10 weeks were more widely distributed and showed a median score shift of two columns to the left on the proforma examination form indicating a diminished reflex response towards 10 weeks (Figure 3). The *compound* score for *reflexes* ranged between 3 and 6 out of 6 in both the 6 and 10-week age groups. Scores between 5 and 6 were found in 86% of infants at 6 weeks and 94% in infants at 10 weeks (Table 2).

### Movements

The *head in prone* item at 10 weeks displayed a wider distribution towards the right from the mutual median RS. This distribution is indicative of increasing postural extensor tone in the horizontal position. The *compound* score for *movements* ranged from 1.5 to 3 out of 3 at 6 weeks and 1 to 3 out of 3 at 10 weeks (Figure 4). The optimal score of ≥ 2.5 was achieved by 94% of infants at 6 weeks and 86% of infants at 10 weeks (Table 2).

### Abnormal signs

The median RS for tremors at 10 weeks shifted two columns towards the left from the 6-week median score, indicating lesser tremor behaviour. The median RS for startle behaviour at 10 weeks demonstrated a one column shift to the right from the 6-week median score (Figure 4). The *compound* score for *abnormal signs* ranged between 1 and 3 out of 3 at 6 weeks and 1.5 and 3 out of 3 at 10 weeks. A *compound* score of 2.5 was achieved by 86% of infants at 6 weeks and 86% of infants at 10 weeks (Table 2).

### Orientation and behaviour

For both items involving visual orientation and alertness at 10 weeks, the median RSs shifted to the right with two columns from the 6-week median score. The median RS for consolability at 10 weeks shifted one column to the left from the 6-week median score (Figure 5). The *compound* score for orientation and behaviour ranged from 3 to 7 out of 7 at 6 weeks and from 5 to 7 out of 7 at 10 weeks. Scores of 5 to 7 were documented in 93% of infants at 6 weeks and scores of 6 and 7 in 96% of infants at 10 weeks. Scores below 5 at 6 weeks and below 6 at 10 weeks were considered sub-optimal (Table 2).

### Total examination optimality score

A total optimality score for the examination was obtained when the optimal scores of all 34 items were added up. The 10th and 5th percentiles were used as cut-off points. In our study, the *total examination* scores for infants at 6-weeks postnatal age ranged between 25.5 and 34 out of the 34 items. The range for *total examination* scores of infants at 10-weeks postnatal age was 29 to 34 out of 34 items. In the 6-week age group, 91% of the infants obtained a *total examination optimality* score between 29.5 and 34. Scores below 28.9 at 6 weeks were considered suboptimal. In the 10-week age group, 94% of the infants obtained *total examination optimality* scores between 31.5 and 34. A score below 30.9 at 10 weeks was considered sub-optimal (Table 2).

## Discussion

Our study aimed to provide a detailed account of the neurodevelopment of 35 healthy term infants, longitudinally documented and scored at 6- and 10-weeks postnatal age, utilising the HNNE to assess 34 neurological items. In contrast to the notable findings of Guzzetta et al. (2005), who identified 6 weeks as a crucial stage for developmental changes, primarily through qualitative observations of neurodevelopmental progress, our study quantitatively evaluated developmental advances using optimality scores across six domains: *posture and tone, tone patterns, reflexes, movements, abnormal signs, and orientation and behaviour* (Dubowitz et al. 1998) in the two subsequent age groups (Table 2 and Online Appendix Tables S1–S6). All the characteristics observed in this cohort of infants at both 6 and 10 weeks during the examinations fell within the range outlined by the standard HNNE proforma evaluation form, confirming the suitability of this tool for use of screening infants’ neurological progress in these age groups. The distribution of RSs and shifts in median scores reveal trends of change, enabling a substantive assessment of infants across these ages. These composite scores can direct clinicians to potential areas of concern or developmental delay. Moreover, *total examination optimality* scores below 29.5 at 6 weeks and 31.5 at 10 weeks can provide a foundational benchmark for subsequent follow-up and referrals.

Key observations underscore the critical nature of neurodevelopmental assessment at 6 weeks, with marked progress by the 10th week. This period shows a decrease in the flexor tone in both upper and lower limbs (lesser resistance to passive manipulation) as well as decreased agility in flexion responses, aligning with the ‘second extensor phase’ of infant postural development (Brown, Omar & O’Regan 1997). An increase in anti-gravity postural control was observed between 6 and 10 weeks, enhancing midline head stability in supported sitting and head elevation in prone positions (ventral suspension and head lifting in prone items – *posture and tone* compound).

This antigravity control reflects the development and distribution of axial extension and flexion control against gravity. These findings contrast with abnormal tone patterns, such as uneven or inconsistent distribution of flexion and extension tone, typically linked to brain lesions and potentially leading to negative developmental outcomes or delays, as discussed in studies by Dubowitz et al. (1999, 2005) and Tuhkanen et al. (2019).

Of interest, phenomena like intermittent fisting with occasional thumb adduction (*abnormal patterns compound*) at 6 weeks can be considered a sign of upper motor neuron lesions if not completely resolved by 7 months (Jaffe et al. 2000). However, in our study, the intermittent fisting and thumb adduction phenomenon were not consistently displayed by 10 weeks as the percentage of infants displaying this phenomenon decreased from 63% to 23%. The scores for this item moved in the direction of more open hand postures at this time point. This characteristic may suggest a developmental transition towards typical hand function, which coupled with a diminished palmar grasp may be influenced by evolving proximal stability and/or positional changes of the head and trunk.

A pivotal development was observed in visual behaviour (*orientation and behaviour* compound), with infants progressing from basic eye tracking at 6 weeks to complete head-turning and sustained attention to visual targets by 10 weeks. This change underlines the critical nature of the 6-week assessment in establishing neurodevelopmental baselines and directional maturation of visual function towards 10 weeks (Mercuri et al. 1999). Focus on a visual target, visual tracking, and alertness are straightforward to observe and assess during initial clinic visits, and even staff with little experience can reliably perform these evaluations (Dubowitz et al. 2005; McGready et al. 2000).

Furthermore, the regulation of behavioural states, as demonstrated through different intensities of *crying* and the *ability to be consoled* co-occurred in brief episodes of increased muscle tone during assessments. Evidence of this, for example, was the *crying* behaviour leading to, for example, clenched fists, underscoring the importance of recognising and understanding the role of behavioural states in the motor performance in young infants (Brazelton & Nugent 1995). At 10 weeks, behavioural regulation showed a wider spread across the five columns, with more infants displaying reduced irritability, an increased capacity for self-soothing, and greater contentment.

At the time of their assessments at 6 and 10 weeks, infants in our study had reached the developmental characteristics identified in the four milestone domains described in RtHB, typically expected by the age of 14 weeks. This emphasises the significance of identifying and monitoring the trajectories of these innate attributes from birth as they progress towards the 14-week milestone.

### Limitations and recommendations

In this study, focusing on a cohort of healthy term infants, the Caesarean section rate was 60%, potentially skewing the sample due to the greater chance of enrolling mothers after extended hospital stay post-delivery. The Caesarean section rates at Kalafong Hospital and the sub-district of Tshwane district is 39.1% and 29.1%, respectively. Our study was conducted solely by one examiner over 9 months; the study acknowledges the HNNE’s high inter-rater reliability of 96% but suggests that an intra-rater reliability calculation be conducted on larger samples of infants for future research.

Despite the limited sample size in our study and the lack of baseline optimality scores of this cohort infants within the first 48 h post-partum, this research offers valuable observations on the neurodevelopmental status and trajectory of infants at and between 6 and 10 weeks, describing important characteristics for the early identification of neurodevelopmental risk. The optimality scores obtained from this cohort advocate for more extensive research and encourage increased proficiency with the HNNE among healthcare professionals like physiotherapists (Narain & Mathye 2019), promoting its earlier use as an effective and accessible instrument for assisting and enhancing child health initiatives within the context of the mother-infant dyad in developing countries like South Africa (Narain & Mathye 2019).

## Conclusion

The milestone characteristics frequently observed in the infants from Tshwane district at 6 and 10 weeks are illustrated by applying the HNNE in our study. Significant neurodevelopmental advancements in posture, head control, muscle tone, and visual behaviour occur between 6 and 10 weeks. By quantifying the distribution of characteristics at 6 and 10 weeks, our study takes an important step in understanding the early traits and trajectory of neurodevelopment in healthy term-born infants within the context of a developing country. Our study challenges the general assumption that little change in developmental characteristics occurs during this early period, providing encouragement and a foundation for future/continuous research in larger cohorts. The findings contribute to the broader goal of improving child health outcomes through early detection and intervention, aligning it with Sustainable Development Goals in South Africa.
